# “I go I die, I stay I die, better to stay and die in my house”: understanding the barriers to accessing health care in Timor-Leste

**DOI:** 10.1186/s12913-016-1762-2

**Published:** 2016-09-30

**Authors:** Jennifer A. Price, Ana I. F. Sousa Soares, Augustine D. Asante, Joao S. Martins, Kate Williams, Virginia L. Wiseman

**Affiliations:** 1 School of Public Health and Community Medicine, University of New South Wales, Botany Street, Sydney, Australia; 2 Ministry of Health, Edifcio dos Servisos Centrais do Ministrio da Saude, Rua de Caicoli, Caixa Postal 374, Dili, Timor-Leste; 3 Faculty of Medicine and Health Sciences, National University of East Timor, Rua Jacinto Candido, Dili, Timor-Leste; 4 Liga Inan mHealth program, Catalpa International, Rua Quinze de Outubro Culuhun, Dili, Timor-Leste; 5 Department of Global Health and Development, London School of Hygiene and Tropical Medicine, Keppel Street, London, UK

**Keywords:** Universal access, Barriers, Timor-Leste, Out-of-pocket payments, Referrals, Traditional medicine, Demand-side, Supply-side, Hospital services, Determinants

## Abstract

**Background:**

Despite public health care being free at the point of delivery in Timor-Leste, wealthier patients access hospital care at nearly twice the rate of poorer patients. This study seeks to understand the barriers driving inequitable utilisation of hospital services in Timor-Leste from the perspective of community members and health care managers.

**Methods:**

This multisite qualitative study in Timor-Leste conducted gender segregated focus groups (*n* = 8) in eight districts, with 59 adults in urban and rural settings, and in-depth interviews (*n* = 8) with the Director of community health centres. Communication was in the local language, Tetum, using a pre-tested interview schedule. Approval was obtained from community and national stakeholders, with written consent from participants.

**Results:**

Lack of patient transport is the critical cross-cutting issue preventing access to hospital care. Without it, many communities resort to carrying patients by porters or on horseback, walking or paying for (unaffordable) private arrangements to reach hospital, or opt for home-based care. Other significant out-of-pocket expenses for hospital visits were blood supplies from private suppliers; accommodation and food for the patient and family members; and repatriation of the deceased. Entrenched nepotism and hospital staff denigrating patients’ hygiene and personal circumstances were also widely reported. Consequently, some respondents asserted they would never return to hospital, others delayed seeking treatment or interrupted their treatment to return home. Most considered traditional medicine provided an affordable, accessible and acceptable substitute to hospital care. Obtaining a referral for higher level care was not a significant barrier to gaining access to hospital care.

**Conclusions:**

Onerous physical, financial and socio-cultural barriers are preventing or discouraging people from accessing hospital care in Timor-Leste. Improving access to quality primary health care at the frontline is a key strategy for ensuring universal access to health care, pursued alongside initiatives to overcome the multi-faceted barriers to hospital care experienced by the vulnerable. Improving the availability and functioning of patient transport services, provision of travel subsidies to patients and their families and training hospital staff in standards of professional care are some options available to government and donors seeking faster progress towards universal health coverage in Timor-Leste.

**Electronic supplementary material:**

The online version of this article (doi:10.1186/s12913-016-1762-2) contains supplementary material, which is available to authorized users.

## Background

Since gaining independence in 2002, Timor-Leste has rebuilt the health system from the ruins of a 25 year struggle for independence from Indonesia. In the first year, the Council of Ministers approved the *Health Policy Framework for East Timor* that was firmly committed to providing free essential services on the principles of equity, population-wide coverage and financial protection, within a highly constrained budget of US$8 per capita per year [1, 2]. The current *National Health Sector Strategic Plan 2011-2030* [3], the recently released 2015 *Comprehensive Services Package for Primary Health Care* [4]*,* the 2010 *National Drugs and Medicines Policy* [5]*,* and the 2007 *Basic Services Package for Primary Health Care and Hospitals* [6]*,* each outline policy initiatives focused on health system strengthening and reiterate the central tenets of free and equitable basic health care aligned with the principles of universal heath coverage (UHC) [7].

The Timor-Leste Ministry of Health (MoH) estimates the public sector delivers about 75 % of total health services via 193 health posts and 66 community health centres across all 13 districts, five secondary referral hospitals and one national hospital; most tertiary care is organised through a limited number of overseas referrals [3]. Mobile clinics use staff from local community health centres (CHC) to provide approximately 450 integrated community health outreach visits every month, locally referred to as SISCa (Sistema Integradu Saude Communitaria) [8]. The deployment of more than 500 Cuban trained doctors has bolstered capacity [9]. Not-for-profit charity and non-government providers supplement public services across the country, while a small number of for-profit private clinics are confined to the major urban areas, Dili and Baucau [10]. MoH guidelines stipulate patients must receive a referral from a primary level health facility before being transferred to a referral hospital, then be referred again to the national hospital, if necessary. This often requires patients to visit multiple facilities before reaching their ultimate care facility.

In some areas, health sector performance has improved significantly in Timor-Leste. Between 2002–12, coverage for a range of essential immunisations^1^ increased from between 14 and 41 % [11] to between 70 and 86 % [12] nationwide. Progress in reducing infant mortality (from 60 to 45 deaths per 1000 births) and under five mortality (from 83 to 64 deaths per 1000 births) surpassed the Millennium Development Goals targets [13]. However, these gains in child health are undermined by poverty and malnutrition. For the same period, stunting in children under 5 years increased from 49 to 58 %, highly correlated to poor maternal health [13, 14]. Also of urgent concern is reducing extremely high rates of maternal mortality. In 2000, at the time of independence, maternal mortality was estimated to be 953 per 100,000 deliveries (uncertainty interval 363–2081), one of the highest rates in the world [15]. By 2010, gradual improvement to 557 deaths per 100,000 deliveries [13] was still more than five times the regional average and double the average for developing countries [16]. Women having at least one antenatal visit had increased from virtually no services in 2000 to 84 % in 2010, yet only 22 % of women were giving birth in a health facility (urban = 52.8 %; rural = 12.4 %) [17]. Accessing delivery care, especially emergency obstetric care in hospital, is the most critical factor in reducing maternal mortality [18]. Both the government and development partners’ strategic plans target the reduction of maternal mortality as a priority intervention [6, 16, 19]. Understanding the barriers to accessing hospital care is essential to achieve this aim and improve health outcomes generally.

In many low and middle income countries (LMICs), poor households are known to face a diverse range of barriers to using hospital services [20, 21]. Demand-side determinants of access to hospital services, such as distance to the facility, poverty, education, opportunity costs and cultural and social barriers, are not dependent on service provision or direct fees for services but significantly affect levels of utilisation, especially for vulnerable groups [20]. In Timor-Leste, the 2014 World Bank *Health Equity and Financial Protection* report found that patients from the wealthiest quintile access hospital care 1.75 times more than patients from the poorest quintile [22]. With 70 % of people living in rural and remote mountainous areas with very poor infrastructure, a quarter of households are more than 2 hours walk to the nearest primary health facility [10, 23]. The 2009–2010 *Demographic Health Survey* [13] (DHS) shows a wide divergence in caesarian section rates performed in hospitals between urban and rural areas. In the capital, Dili, the caesarean rate of 4.8 % compares to 0.1 % in Ermera district (national average 1.5 % of total births). Other case studies from Timor-Leste demonstrate the complex interplay between socio-economic determinants (influencing demand) and weak health systems (influencing supply) on access to health services [24, 25]. For tuberculosis (TB), the second highest cause of death in Timor’s hospitals [3], treatment adherence was “influenced by a complex interaction of structural, personal and health service factors operating within a social context” [26]. On the supply side, health seeking behaviour and uptake of services are adversely affected by poor service delivery, negative staff attitudes shaming patients and lack of human and material resources [27, 28]. Women wanting to give birth in a facility are primarily concerned that qualified staff and medicines will be available [13, 29]. Equally, overcoming the stigma of having TB and receiving reimbursement for food and travel costs were identified as important incentives for persuading TB patients to continue regular hospital visits over several months [26]. How these various demand and supply-side factors directly influence hospital utilisation rates in Timor-Leste has not been investigated, despite evidence of highly inequitable utilisation of hospital services.

This paper investigates the reasons driving the inequitable utilisation of hospital services in Timor-Leste. To do this we employ a conceptual framework developed by Peters et al. [30] and later adopted for use by others [31]. In this framework, four dimensions of access are identified: geographic accessibility (defined as the physical distance or travel time from service delivery point to the user), availability (having the right type of care available for those who need it as well as having the appropriate type of service providers and materials), financial accessibility (removing financial barriers to health care and protecting citizens from catastrophic health expenditures) and cultural acceptability (defined in terms of how responsive health service providers are to social and cultural expectations of individual users and communities), with ‘quality of care’ underscoring each dimension. Each dimension includes factors that influence the demand for health services within a community or household, and supply-side factors that may be influencing service delivery [32–34].

In this study we investigate how personal experiences accessing hospital services have impacted on community attitudes to seeking hospital care and identify barriers that need to be addressed. Institutional responses from Directors of community health centres dealing with the constraints of the primary health system and initiating hospital referrals are also analysed. References to relevant policy interventions and recommendations are included in the final discussion. Within this context, the aim is to better understand why people in Timor-Leste and other countries are prevented, or choose not to, access hospital care, significantly inhibiting progress towards the shared goal of improved health outcomes through universal health coverage – defined as access for all to appropriate promotive, preventive, curative and rehabilitative services at an affordable cost [35].

## Methods

This study was designed as a multisite qualitative study using focus group discussions (FGDs) and in-depth interviews (IDIs). In August 2014, we conducted eight FGDs, segregated by gender in eight districts of Timor-Leste. We also conducted eight IDIs with the Director of each CHC in the same districts where the FGDs were held. The locations were purposively selected to reflect conditions in the 13 districts of Timor-Leste, including urban, rural and remote settings, across different demographic groups. With approval from the MoH, a local collaborator of the study contacted each community informing them of our intention to conduct FGDs, the aims of the research and how the information would be used. No criteria were imposed on the selection of participants, except being over 18 years, living locally and interested to discuss their experiences accessing hospital services.

The FGDs were held at a local venue with 6–8 participants (*n* = 59; 22 male, 37 female, age range 19–75 years), moderated and recorded by an experienced Timorese health researcher (AS), speaking Tetum, with an observer (KW) taking notes on non-verbal responses and underlying themes. This arrangement was decided upon following a pilot FGD that trialled using both moderators in the discussion; one directing the discussion in English and the other translating the questions and engaging the group in Tetum. Both the participants and moderators found this approach inhibited discussion. Using the discussion guide and an experienced local moderator (AS), it was decided to have one moderator lead the discussion and another as observer. Another change following the pilot FGD was to move from mixed-gender community groups to gender segregated groups. Every effort was made to help respondents feel relaxed and secure in expressing their opinions, minimising interviewer effects [36]. The study was approved by the Human Research Ethics Committee (HC13269), University of New South Wales, Sydney, Australia and by the Ministry of Health, Timor-Leste. All participants gave written informed consent before being interviewed.

The FGDs and IDIs were conducted using a pre-tested semi-structured interview guide beginning from a broad perspective (understanding the purpose of having a health service) to individual experiences accessing hospital care and those of people they knew, how those experiences influenced their attitudes and willingness to seek health care in the future and suggestions for improving services. The IDIs focused on critical challenges providing services (patient transport, medications, equipment), experience using the hospital referral system (use of official referral guidelines, reasons for referring in practice, whether patients use their referrals, followup) and recommendations for improving service delivery. (see Additional files 1 and 2)

The moderator (AS) and observer (KW) simultaneously translated and transcribed the recordings to English, then the moderator and another author (JP) independently coded the data using best practices guidelines [37] to develop themes and emerging concepts (Table 1). The conceptual framework was then used to group emergent themes according to the dimensions of geographic accessibility, availability, financial affordability and acceptability (see ‘Background’ for a description of the conceptual framework) (see Table 2).

**Table 1:** Coding procedure and evolving research analysis

	Action	Development of case study ideas
Raw data	8 FGDs – gender segregated; urban and rural 8 IDIs – Directors CHC, urban and rural	Hospital utilisation pro-wealthy Determine if barriers to hospital care exist.
Coding	Code data (interviews) by topic, frequency, sub-group (gender, rurality)	Descriptive coding to identify barriers: individual, community, health facility.
Themes	Group codes into categories	Develop coding framework: group consistent and related themes to identify systemic barriers into categories.
Concepts	Investigate relevant conceptual frameworks, related research; select analytical framework	Align coding categories to Peters et al. [29] framework of access criteria: geographic accessibility, availability, financial affordability, social acceptability.
Theory	Universal health care requires universal access: determined by health and non-health related factors.	Case study demonstrates direct costs for health care are only one factor determining access. Demand and supply side interventions are needed to address barriers.

Adapted from Chandler et al. [36]

**Table 2:** Summary of barriers to accessing hospital services in Timor-Leste

Geographic accessibility	Availability	Financial Affordability	Acceptability
Supply-side factors *Service location* Hospital coverage - long distances to hospital - 13 districts, only 6 hospitals - 25 % patients >2 h to primary facility Patient transport provided by health service - cannot access all areas - limited availability during wet season - no service to transfer patient home - recovering patients stranded Demand-side factors *User’s location* Isolated communities - Infrastructure poor - rugged terrain, poor roads + bridges - ambulance cannot reach patient - patient journey to services difficult Public transport - no connections to distant villages - infrequent services Private/community transport - uncomfortable, difficult journey being carried the ‘traditional way’ - walk, use porters, horse - unreliable, ad hoc arrangements - family/community vehicle, police - need to hire private car, truck, motorbike	Supply-side factors *Health workers, drugs, equipment* Patient transport - too few ambulances - lack of coverage - poorly maintained - out of service, no fuel, no driver *Service delivery* Short opening hours - unable to access services after 3 pm - facility phone not answered 24 h Ambulance not available 24 h - no emergency service outside hours - no referral to hospital outside hours Long waiting times at hospital - staff not assisting lost patients - randomly rescheduling appointments Laboratory tests, blood supplies - few service locations - service availability erratic Medicines – regular stockouts *Human resources* Staff often not available to accompany patient in ambulance - rely on companion for clinical support Demand-side factors *Patient transport* Fear of being stranded after hospital visit - no fee support for transport home - patients stranded while still in recovery Repatriating deceased relatives - limited provision through health services - no established private providers	Supply-side factors Medicine stockouts in public sector - purchase medicines from private sector Blood supplies limited - high cost of blood from private donor - no standard charge Demand-side factors *Costs and prices of accessing services* Out-of-pocket expenses - Ambulance - patients/family pay for fuel - patients/family pay driver - costs to return home after transfer Transport charges - private car, truck, motorbike - public transport fares - repeat visits extra burden Indirect costs - food, accommodation, transport - recuperation period - companion/s costs - gifts, contribution to host family - opportunity costs - lost income - divert money needed for essentials Repatriation of the deceased - provider surcharge to carry deceased - large families, costs multipled - previously experienced prohibitive costs - families choose to not seek care - reserve money for funeral costs *User’s resources and willingness to pay* Ability to pay limited, poverty rate high - 44.3 % population below US$1.25 per day - direct + indirect costs barrier to access	Supply-side factors *Characteristics of health services* Staff conduct - blame and shame attitude to vulnerable - shouting at patients - delaying care, prolonging labour Nepotism - ignoring patient requests for assistance - fast track wealthier patients, family Provision - service coverage poor - six hospitals to cover 13 districts -laboratory tests - irregular availability - patient transport poor quality Demand-side factors *User’s attitudes and expectations* Dissatisfaction with quality of services - fear journey/transfer to hospital without medical supervision - disrespectful staff attitudes, nepotism Social isolation visiting hospital - hospital far from home, unfamiliar area - overwhelmed by hospital systems - depend on family support near hospital - resignation/preference to ‘die at home’ Medicines - unconvinced medicines effective - given same medicine, different illnesses Traditional medicines - strong cultural belief supports efficacy - acceptable substitute/preferred option - used to complement medicines

Adapted from Peters et al. [29]

## Results

Both the FGD respondents and CHC Directors identified chronic shortages in patient transport as the principal barrier to accessing hospital care in Timor-Leste. The multiplier effect from this one factor is considerable: delays in accessing care, significant out-of-pocket expenses for private transport, increased health risks for patients transported without clinical supervision or basic equipment, and providing shared stories within the community that act as a strong deterrant to others from seeking care. In many cases, the cost and trauma associated with accessing hospital care was compounded by additional out-of-pocket expenses and low standards of professional care once they reached hospital. While CHC Directors were understandably cautious criticising the health system, FGD respondents spoke candidly about their experiences and how they negatively influenced future decisions to seek hospital care. Although the question guide for the FGDs did not specifically ask about hospital access to treat certain conditions, two issues dominated discussions. First and foremost, very significant and intractable barriers for accessing emergency obstetric care, and secondly, the difficulties faced maintaining long term repeat visits for chronic diseases, such as tuberculosis. The government’s policy commitment to provide free basic quality health care has built a level of expectation within the community that is not being matched by the current level of service provision. A summary of findings is presented in Table 2.

### Geographic accessibility to hospital services

Any discussion regarding barriers to accessing hospital services in Timor-Leste must appreciate the impact of the rugged mountainous terrain, with extremely poor standards of infrastructure, especially for remote communities. With 90 % of roads rated as poor or very poor [10], landslides and swollen rivers during the wet season that make roads and bridges impassable, conditions severely restrict access for patient transport to reach the highly dispersed villages and for patients to travel independently. Both the respondents and the Directors of CHCs cited distance and transport difficulties as the most common reasons for patients not seeking care; one-quarter of households are more than 2 hours walk from their nearest primary facilty [27]. Average travel times to reach one of the six hospitals that cover the 13 districts are not available. However, given the rugged conditions, low levels of transport infrastructure, patient transport and hospital coverage, geographic inaccessibility is the major barrier to reaching hospital for the majority of people.

Most rely on family and neighbours to carry them ‘the traditional way’, such as an open cart known as a gerobak.

*“We tried to call the ambulance but the driver replied that the ambulance was broken. We were suspicious that the ambulance did not come, not because it was broken but because the road condition is bad and our village is far from the CHC. Anyway, the patient had no strength to do the delivery at home, so the families decided to take her to CHC by gerobak pushcart. Because our village is far away and the baby is also bouncing, so the baby was born inside the gerobak on the way to the health centre.”* [Urban female, 35]

*“… sometimes when it rains the trees will collapse over the road, sometimes it gets cut off, this makes it difficult for us when we have to refer patients. This road was built during the Indonesian times, there are parts that were rehabilitated but then destroyed again by natural causes.”* [Rural D-CHC]

From one remote area, patients were carried 16 km to the nearest health post, travelling for 9–11 hours.

*“We start walking at 3 am and arrive* [at the health post] *12 midday. Therefore most pregnant women have the delivery process at home.”* [Urban female, 47]

Using whatever transport options are available to reach care, patient distress and adverse clinical outcomes during the journey was a regular theme in FGDs.

*“A pregnant lady received a referral to hospital yet the ambulance service was not available. She had to travel to hospital in a truck. On the way to hospital, she had to do an immediate delivery process in the truck. Unfortunately, the delivery process failed. The mother and the baby passed away during the process. Hence, we had to carry the body back to her house because the truck would not bring back the cadaver. People believe that public transport* [for business] *cannot take the cadaver because it will bring disaster to the company.”* [Rural male, 31]

Finding transport to hospital is a primary deterrent for the elderly and their family deciding whether to seek care.

*“Some of my neighbours, particularly the elders, could not make it to the health centre or Dili hospital for treatment because they cannot walk. They hear some bad experiences from other patients.”* [Urban female, 44]

Similarly, the CHC Directors (D-CHCs) consistently reported difficulties accessing patients living in remote areas, having unsuitable vehicles for the rough conditions.

*“First of all, the road in the villages is still a problem. The Ministry of Health, when allocating the transportation such as the Ranger model, it’s not conditioned to the type of terrain. Only cars like the Hilux or Highlanders with bigger tyres can operate in this type of territotry because the road in our rural areas are unpaved and when it’s raining, it’s really difficult for us.”* [Rural D-CHC]

Most of the CHCs relied on a ‘multi-function vehicle’ to transport critically ill patients, although both patients and the Directors agreed, it is not what they are not designed for.

*“Sometimes the hospital ambulances are all occupied so then the multi-function has to transport the patients who we referred. That is what we are implementing so far and about the equipment that we use in the multi-function, they are not adequate. So it makes it difficult for us. …the multi-function is not made for transporting people because there’s nowhere the patients can lie down in there because it is not equipped as an ambulance would be. The car is made for people to sit in, not lying down. And sometimes the oxygen that they prepared in there, sometimes* [there is] *only empty tanks. So when we transport the patients we have to create conditions for it so this is what makes it difficult for us, that we need to improve.”* [Rural D-CHC]

Each CHC endeavours to use whatever transport is available, either from the neighbouring health facilites -*“To be honest, our multi-function* [vehicle] *operates for 1 or 2 weeks and then it breaks down so when it is in this condition, we will contact* [the neighbouring CHC]” - the police or one CHC had made a special arrangement with a local NGO.

*“If the condition of the vehicle is good, we feel we can refer more. But… sometimes we call and the car is somewhere in the rural area, we feel that this is also a difficulty because sometimes there are patients who are on transfusion and in need of urgent transfer, or bleeding. When during that time all the cars are being used, then it’s a problem. Sometimes we will contact for help from NGOs or the national hospital so they can send an ambulance to transport the sick… We discussed it at the coordination meeting, that we have difficulties during the referrals.* [We asked the NGO] *‘If we don’t have any transportation available, can we contact you to come and help us?’ and they said ‘Yes’.”* [Urban D-CHC]

Ultimately, the CHC response is highly dependent on the prevailing sentiment of the Director and staff, the frustration is evident. One Director announced he had stopped staff using the multi-function vehicle to transport patients to hospital, on principle*“Now that I have prohibited it, they don’t do it anymore… the hospital must come to pick up the patient from the health centre.”* Another was more amenable to local challenges:

*“For the patients themselves, with the difficulties they have, they couldn’t come to the health centre, therefore we…come to them when the family or community members have informed us about a patient. So in this referral system, we have to come to them because they don’t have good access to road, transportation,…” *[Urban D-CHC]

### Availability of hospital services

Unlike the barriers imposed by geographical inaccessibility and weak transport infrastructure, inadequate coverage and malfunctioning ambulance services are the responsibility of the health system. Complaints about the lack of availability of ambulance services were mentioned in every FGD and by each Director: long delays because the patient transport was on another call, not available out of hours, out of service, having no driver or no fuel. Several patients who managed to obtain transport through the health service reported being transported without a staff member, relying on family or friends to care for them. However, Directors of the CHCs interviewed reported that medical staff were always available to accompany patients being transferred to hospital.

*“…if we arrive here* [at the CHC] *and the health workers transfer us to* [the referral hospital] *we always have difficulty with transport. So we have to organise private or public transport. The ambulance is always broken, or there is no fuel, no driver because the driver has died. The driver passed away 8 months ago and hasn’t been replaced. … We know it is impossible to get transferred… They take our IV out and we go* via *public transport. Even if an ambulance does take us, there is no health worker with us. We just go with a family member and driver.”* [Rural female, 24]

*“I went to Dili hospital* via *ambulance. At first I couldn’t though as the ambulance had no fuel. This is a big problem and happens many times. Finally they got some fuel. At the time I was unable to walk or sleep well and the ambulance had no mattress and was in a bad condition. Then they got a mattress and took me. I arrived at the ED* [emergency department] *and they pushed me* [via wheelchair] *to registration and registered my name.”* [Urban male, 55]

Confusion about how to find the services they need at the hospital, the procedures to receive treatment (such as announcing your presence to reception) and long waiting times (sometimes being asked to return the next day) caused some respondents to dismiss the value of visiting hospital and receiving treatment, deciding to use traditional medicines instead.

*“This is my first time at the hospital and I don’t know exactly which place to go to but they didn’t care about me… I waited and waited with no one caring about me. I returned home …Why come back? I went to a private pharmacy and got the medicine instead…I want to say again, when I went to the hospital they were rude to me. I don’t like to return to that hospital and I also have some traditional medicines.”* [Urban female]^2^

*“These health service systems are confusing…If we arrive at 3 pm then they say ‘you are late’, doctor is not here, you can go back and return tomorrow. This disappointed and annoyed patients who come from far villages like us. Consequently, some patients do not want to go back to hospital but chose to return home to the district.”* [Rural male, 29]

Directors of CHCs agreed that many patients and their families hesitate and often find excuses for not attending hospital, or lose their referral.

*“… some patients who have understanding about the referral, they will come but if they are older people, for example, they will have a lot of excuses, such as ‘Who is going to accompany us at the hospital? Who is going to look after the children?’ and stuff like that. That’s why they would not come to the hospital but the staff always tell them that they can come to the health centre first for observation and if the doctors approve, then you will just receive medication and can come back home, so no need to go to hospital. So we always try to give them positive thinking so they can come. I think the doctors and nurses have ways that they can convince their patients.”* [Urban D-CHC]

*“… sometimes the patient doesn’t want to go* [to hospital] … *They said, wait let’s communicate with the patient’s family; so many times we saw they lost the referral letter. So that always happens in the centre, … some we give them referrals today, they will come up with a lot of excuses and lose the referral. Some, they got to the hospital but don’t go in as soon as they see the situation there. There are a lot of people at the emergency so this becomes a challenge for them, so they decide all of a sudden not to go in. So we don’t know where they ended up, even though we have their record of registration here at the centre but we don’t know where they’re from, where do they live, because we cannot control all this.”* [Urban D-CHC]

Participants felt strongly that assistance with the return journey should be included as part of the continuum of care, based on their level of need.

*“…the ambulance needs to take us back. That’s why we don’t want to go there… It is difficult to go out and try and get public transport because we are walking like drunk people, we are 100 % still weak. It would be better if they came and got us.”* [Urban male, 64]

### Financial affordability of accessing hospital services

Out-of-pocket expenses incurred by patients and their families when accessing hospital services cover two broad categories, i) subsidising the shortfall in public health services including patient transport, medicines, blood supplies and repatriation of the deceased, and ii) indirect costs for the patient, their family and companions including food, accommodation, travel expenses and lost income.

The 2014 World Bank study on health equity and financial protection in Timor-Leste found user fees represent only four percent of total health expenditure, with minimal adverse effect on households’ level of poverty or impoverishment [22]. However, results from this research suggest total out-of-pocket payments by the patient and their families for travel costs, companion costs and health related costs (medicines, blood supplies and repatriation of the deceased) places a serious financial burden on these households. Considering 44.3 % of the population earn below the poverty line of US$1.25 per day [22], respondents in the FGDs felt paying even small amounts of money for public transport, food and medicines from private pharmacies (especially repeated costs for chronic illnesses like TB) were preventing them accessing services or maintaining their treatment regimen. Large amounts for one off payments, such as emergency private transport, repatriation of the deceased or sourcing private blood supplies, were prohibitively expensive, causing many to not seek care.

#### Transport costs

Timor-Leste’s 1.1 million people are highly dispersed through rugged, mountainous terrain affected by flooding and landsides in the wet season. Over 90 % of roads are rated as poor or very poor, with extremely low coverage for the 70 % of people living in rural areas [10]. When public transport was available, repondents generally paid between $2 and $10, forcing some to borrow money to cover the cost.

*“Every Thursday I had to go to the hospital to have treatment… During this 2–3 years I went only twice with the ambulance … An anguna* [minibus] *is $2 to Dili. If I have no money, I have to borrow. I will say to the driver I will pay when I get home, hoping my family will have found some. But if I have no money I can’t go. Sometimes the driver will let me pay later. If he says no, I can’t go.”* [Urban male, 60]

*“It becomes an issue that will prevent us from doing our next check–up in* [the referral hospital] *or Dili. We have no choice, because we want to get better from our sickness so… we have to try hard to get money, otherwise we borrow our neighbour’s money to do the treatment.”* [Urban female, 44]

Charges for hiring private transport were consistently reported between $50 to $100 for a one way trip to hospital.

*“In my village there is no public transport to rent particularly in the night. Finally, my husband called my brother living in town to help us find transport to pick me up and take me to the hospital…For those who have a serious illness or pain, they must be carried by family until they pass the river, then look for and hire transport - truck $50 and mikrolet/anguna minivans $100 dollars - to bring the patient up to the CHC. Because of the road conditions… cars rarely want to get there, even if the patients are able to pay the high price. For those who don't have money and suffer severe disease, they’re just waiting to die."* [Urban female, 36]

Respondents often reported opting out of care because of prohibitive transport costs.

*“My father and I had to stay in Dili for 2 weeks. Because my father is sick, we cannot travel in the mikrolet* [minibus] *to hospital… The taxi fee is so expensive, we have to pay $8 for round trip. We have high expenditure during the treatment in Dili, consequently we were running out of money and we decided to return* [home]*”* [Rural male, 34]

#### Blood supplies

In 2012, a Red Cross situational analysis of blood supplies in Timor-Leste found 2400 units of blood were needed for transfusions but only 1938 units (81 %) was collected, mostly replacement donors from family members of patients that require the transfusion [38]. Community mobilisation for blood only occurs in Dili district, and blood transfusion services are only available at the five district referral hospitals and the national hospital in the capital, Dili; seven districts have no blood transfusion services [39]. For elective surgeries patients are asked to bring their own donors and in an emergency, blood is often collected from family, friends, the police and military personnel [38]. In the FGDs, several respondents described their family’s desperate search for blood, eventually paying between $30-100 per bag from private individuals, including from family and friends, if no other supply was found.

[After a complicated birth delivering twins that later died, the patient was transferred from the CHC to the referral hospital.] *“The doctor* [at the CHC] *helped take out the placenta. They said I had no blood and they transferred me* [to the referral hospital]. *They said we needed to find blood. We have no family there and don’t know how to do this. My husband asked people in the hospital. We found them and we had to pay $30. They were my husband’s friends… After 3 bags were finished they said I was still anaemic and they transferred me to Dili. …In Dili we have no family and again they said we have no blood. We went to FFDTL* [military base], *they came to the hospital but unfortunately they were not the same. During the hospital stay one doctor came to me and I didn’t recognise he was a family member… He donated and I didn’t have to pay him. Then he looked for 3 more people. I then had to pay $30 a bag again, costing $90. I stayed 1 month in hospital.”* [Rural female, 27]

*“Since there is no blood supply, the patient's family ran around looking for donors and the patient died due to running out of blood. When I was hospitalised my family bought three bags of blood; the price was $300* [$100/bag].*”* [Urban female, 28]

#### Repatriation of the deceased

Another major expense for respondents was the cost of repatriating the deceased, particularly the elderly. Since 2009, the government has had a policy to provide funeral cars [40, 41] through the Ministry for Social Solidarity, however FGD respondents did not mention the policy nor having benefitted from it. Instead, many respondents mentioned the difficulties involved organising private arrangements, especially because of the commonly held belief that transporting a cadaver brings bad luck, increasing the cost of hiring transport, beyond the reach of many.

*“Three incidents happened to our family. Our 3 children passed away … So as ordinary people, we cannot do anything to cure our child and the child has to die. As part of that, when our child passed away at* [the referral] *hospital the ambulance cannot evacuate the dead body to remote villages. Hence, we have to hire private ambulance and fill it with fuel which cost up to $100/trip… Based on this experience, we decided not to bring our fourth child to* [the referral] *hospital and we prefer they die at home than suffer like the other three family members.”* [Rural male, 42]

*“The oldest people in the village are afraid… When they pass away no-one can pay to have them taken back… like $250. So it is better they stay here and not put more pressure on the family… Better to just die here. We want to go to Dili because we want to survive but it is hard.”* [Rural female, 27]

Being unable to afford a funeral car, FGD participants spoke of families carrying their deceased home, on a motorbike or family members carrying them home.

*“One day a baby died in the referral hospital and the family had no money to pay for gasoline ($15), therefore the family had to bring the cadaver back home by motorbike. Last month there was also a young male patient who died in a referral hospital after midnight, around 1 am. … the funeral car was damaged so the families had to carry the cadaver home* [far from the hospital]*. Transportation will not be rented to transport the cadavers because there is a traditional belief that will bring bad luck to their business.”* [Urban female, 38]

Faced with the difficulties and expense of repatriating the deceased, some respondents prioritised the traditional village burial over seeking hospital care.

*“We do not want to go to* [the referral hospital] *or Dili because we prefer to save money to buy coffee and tea for the funeral preparation than pay for transport fuel.”* [Rural male, 69]

*“The family had no money to bring the baby back. The health worker said ‘the body is going to be in the land here or there, so why not here. The mother is still stressed as a result.”* [Rural female, 35]

*“… when the patient dies, no car transfers their body back home. Therefore, they just surrender to their diseases and say ‘I go I die, I stay I die. Better stay and die in my house and not put many pressures or make trouble for the families.’”* [Urban female, 44]

#### Food and accommodation costs

Respondents were acutely aware of the cumulative costs accrued during the time spent away from home, particularly paying for food for companions. Patients’ food would be shared with the companions who slept at the hospital rather than incur extra costs.

*“In hospital the food and bed are provided only for the patient, and the person who accompanied the patient did not get food and a bed inside the room/hospital. Therefore family members had to buy their food outside. During treatment… we did not buy any medicine, all medications were provided. The only difficulty is about the food for the family members who look after the patient.”* [Rural male, 24]

“*I have another experience when I stayed in hospital. I see with my own eyes where the families divided a plate of food (actually for patient) into two parts so that both the patient and caretaker can eat a little bit.”* [Urban female, 36]

A constant theme was whether there was family in the vicinity that could support their visit; close enough to the hospital for affordable travel costs and to provide accommodation for the companions and the recuperating patient after their release. FGD respondents did not mention paying for commercial accommodation. Rather, without family, patients are unable to access hospital care and others returned home before care was completed.

*“Apart from transport, expenses for food is another issue for us to travel to* [the referral hospital] *and Dili. For patients who do not have family in Dili then he has no reason to stay in Dili. The patient will decide to return… home because they have no family to look after him/her.”* [Rural male, 34]

*“… we stay in Dili with our family but we also have to help them, such as share to buy some food and credits for electricity* etc.*”* [Rural male, 29]

### Acceptability of hospital care

Complaints expressed in the FGDs regarding quality of care highlight patients’ sensitivity to negative responses from hospital staff, especially for patients dealing with new surroundings, unfamiliar treatments and a sense of vulnerability. Some stories shared in the FGDs were from personal experience and others were repeating what they had heard in their community: negative experiences are shared with powerful effect once patients return home. From the FGDs, poor quality care fell into two broad categories, i) poor clinical service due to an under resourced health system, and ii) poor professional standards of care from staff and unfair selection processes for receiving treatment. Similarly, the Directors of the CHCs were frustrated by not having adequate resources for basic equipment, such as weight scales, blood pressure gauges, stethoscopes and basic testing kits, limiting their clinical scope and prompting avoidable referrals to hospital care.

#### Availability of medicines and diagnostic tests

Patients reported visiting referral hospitals for tests that were previously available but were told they now needed to go to the national hospital or pay for the test from a private laboratory. They were confused and suspicious that the services were being withheld.

*“The last 6 months when I went there they did this examination for me. I don’t know why they said this time that they have no examination for urine.”* [Urban female]

Needing to access the national level services when district hospitals were unable to deliver basic services meant some patients failed to attend follow-up treatments and tests.

*“During this 3 months, I am doing* [TB] *treatment in* [the referral] *hospital. There’s now an X-ray test for sickness at* [the referral] *hospital.… The doctor recommended I do x-rays in Dili. I stayed in Dili for 3 days and the x-ray result is that I suffer from TB …The hospital asked me to return to Dili hospital for check-up … but I could not make it for the check-up because I have financial issue that prevent me returning to Dili.”* [Rural male, 66]

Not being able to access medicines at the local facility that the patient believed would treat their illness caused many to resort to traditional medicines that were available locally.

*“I think both traditional medicines and other medicines are the same. They both have advantages and disadvantages… we have to help ourselves. This is our culture when we use traditional medicines to help cure us.”* [Urban male, 75]

*“When we use the traditional medicines and we recover we don’t want to come back to the clinic … Sometimes you come to this clinic and I receive the same medicine as the time I came with a different illness. I don’t want to come back. I prefer traditional medicines.”* [Urban male, 66]

Directors agreed that there were persistant and long term stockouts of essential medicines, sometimes for months at a time, including for the most basic items such as antibiotics, paracetamol, ibuprofen, iron supplements and oxygen for the clinic and patient transfer vehicles. Lack of medicines is a central complaint from both the clinical and patient perspectives. Several Directors mentioned that the recent initiatives to decentralise the supply chain for pharmaceuticals had not improved the situation, making it worse in some instances.

“*At the moment we have a pharmacist but the person doesn’t come in to work. Usually we make an emergency request 2 months before we run out of the medication. But every time we do that, they always fail to send us the medication as we requested. So I would like to ask the Ministry of Health to pay attention to this because when there’s no medication, they probably don’t feel anything but it’s a problem for us who work at the community level because we don’t know how to explain it to the patients. Sometimes they come to us every 2–3 days, we try to expain it to them, about the situation. Some patients they understand about the situation but some won’t understand. They told us maybe* [it’s] *better* [to] *shut down the health centre, better shut down the hospital because it doesn’t have any medication.”* [Urban D-CHC]

In response to the constant stockouts, the Directors said they try to substitute another medication with the best clinical fit, prioritise patients (usually pregnant women and children), dispense a placebo such as a multi-vitamin, or reduce the dose as far as possible. Some felt the newly decentralised distribution sytem that depends on district authorities to sort and allocate the products for each facility is causing avoidable delays.

*“Our recommendation is to go to the first government system when… we received medication in packets. It’s already divided* [at the national centre] *for each village, they have their own packet labelled with names… so we don’t have to wait until it gets to* [the district facility]*… If the Ministry of Health could figure out a way to implement that again, it will be better, the first government system.”* [Rural D-CHC]

#### Poor professional standards

Vivid stories of wrongful treatment in some hospitals were reported by many respondents. Although it is not possible to judge clinical accuracy, the themes were consistent; women in hospital with prolonged labour left unattended, sometimes for days, ending in maternal and/or infant mortality; angry and insulting remarks from the staff; being ignored and passed over while waiting for treatment; lack of assistance finding the correct service delivery point; and confusion about how to access services.

*“During the process of delivery we were crying because of the pain but the midwife was very angry and said, ‘you guys don't yell when receiving big banana but now you want to give birth, you shout.’”* [Urban female, 27]

*“A pregnant woman from our village had signs of labour. The patient went to the hospital and stayed for three days and three nights. The patient felt so much pain but the doctor and midwife asked her to continue to hold the pain. On the fourth day, the doctor said that patient would have surgery. Unfortunately during the operation the baby died in the womb but for three days in pain the baby moved, it was so active.”* [Urban female, 42]

*“It has occurred in our referral hospital here. The health workers told patients not to yell during the process of giving birth and withstand the pain until the morning. In the morning when the doctor and midwife came to help with the birth, the baby’s heart rate no longer functioned and had died. After this, news is spreading and circulating around this district, the pregnant women in our village would not come to give birth in hospital but they choose and prefer to give birth in their hamlet/village assisted by a traditional midwife.”* [Urban female, 26]

*“In the middle of the night the infusion got stuck and caused swelling. Our family immediately reported it and called the health workers, but instead we got yelled at saying ‘Are you guys dogs or humans?’. “*[Urban female, 28]

*“Health workers yell at us like a slave … they give priority to the important people, rich and intellectual and neglecting the poor, no money, stupid and dirty…That is the reason why people do not want to go to the hospital although they have a letter of referral.”* [Urban female, 26]

Some of the Directors interviewed acknowledged they had heard reports of patients receiving unprofessional or abusive treatment but did not delve into the issue. However, one Director did suggest that staff being rude with patients can be an expression of their frustration when attempting to educate patients.

*“Well, sometimes staff don’t quite have the ethics because the tone that we use when talking to the patients is sometimes rude. But from our perspective, we use* [that] *tone because we want to educate them. Because we see people’s characters, sometimes they say yes, yes, but in reality they don’t do as we told them. So then it looks like we don’t show good ethics when sometimes we yell at them but sometimes it’s because it’s difficult for the community to change their behaviours.”* [Urban D-CHC]

Based on the FGDs and from the Director interviews, most reports of denigrating staff behaviour and verbal abuse related to hospital facilities (rather than CHCs), particularly from hospital midwives. One Director mentioned this specifically.

*“In* [the regional hospital] *I know that the attitudes of the midwives are not good. Many people in* [that town] *prefer to come to the sub-districts because it’s better in the sub-districts, they attend to people who give birth better. In* [that town]*, there are one or two midwives who don’t attend to people very well, they are very rude. Usually they react badly to people who scream by uttering bad things, that is why many people don’t like* [them].*”* [Rural D-CHC]

For many FGD participants, lack of familiarity and bewilderment with impersonal hospital systems and a sense of social isolation were strong deterrents to accessing hospital services.

*“This is my first time at the hospital and I don’t know exactly which place to go to but they didn’t care about me… I waited and waited with no one caring about me. I returned home …Why come back?… I don’t like to return to that hospital.”* [Urban female]

*“Please do not send and leave us alone with a piece of paper, particularly for those who have no families in Dili and no money, because staff in Dili hospital will not take care of us. ”* [Urban female, 36]

Community members and CHC Directors agreed that often family connections were essential to receive the best care and socio-economic status strongly influences the level of attention and respect the patient receives.

*“When we go to the hospital there is a queue outside, but others go through the back to see the doctor if they are family of health workers. This system disappoints us, the patients who have no relatives inside the hospital. This system is preventing us going to hospital…So terrible, I hate this system.”* [Urban female]

*“They were disgusted by us because our life is simple and dirty… When they examined the patient* [their grandmother] *they clamped her shirt with two fingers* [participant demonstrated how to clamp with the thumb and index finger with the right hand, left hand covering mouth and nose] *while tossing bad words like she was dirty…In that time there were patients who came by hartop* [car] *and motorbike. The health workers directly served them well and smooth.”* [Urban female, 28]

*“We use* [verbal] *force and threaten and say, ‘If you don’t help us we are going to parliament.’ You see inside the room they are just sitting there laughing and talking and we have to be kept waiting. ”* [Urban female]

Nepotism was mentioned, either directly or implicitly, by most CHC Directors as a critical systemic problem, despite government policy and public support for equitable access to health care services, including hospital care. Several Directors spoke passionatey about the need for fairer treatment and better access for the vulnerable.

*“If you know someone well at the hospital you will be preferenced. If you don’t have family or connections then you will be abandoned… For people who don’t have knowledge about health services, and no family or connections, that’s even worse. People with higher education, who have knowledge about many things, they go to the hospital and already receive such treatment. How much worse is it for people with no education background? Especially if they don’t know how to read, write or speak; they will be left unattended.”* [Urban D-CHC]

## Discussion

Improving physical access to health services in Timor-Leste has been prioritised in several strategic analyses [3, 6, 10, 16, 42]. Policy responses focus on improving the supply of health services, including integrated community outreach and improved primary health care [4]; increasing the stock, maintenance systems for ambulance services [16]; and improving the collection and coverage of blood supplies [38]. A financial manager and a long-term supply chain manager have been appointed to strengthen the supply and distribution of pharmaceuticals [43]. A m-Health project linking pregnant mothers with health providers using mobile phones is showing promising results, increasing demand for facility births [44]. Although these interventions are designed to improve access, research findings for this paper suggest barriers to accessing hospital services, specifically, are more complex, onerous and deeply embedded. Policy makers, donors and other stakeholders designing and implementating changes to the supply of health services and wanting to increase utilisation would benefit from researching supply and demand side factors identified in this paper.

There are more innovations in financing, service delivery and regulation of care that hold promise for improving access for the poor and vulnerable [30, 32]. Evidence from other LMICs show improved ambulance services, including motorcycles as a cost effective option for reaching inaccessible areas, including for emergency obstetric care to prevent maternal and neonatal deaths [45–47]. Similarly, for patients with a chronic illness who need to make regular trips to hospital for testing and treatment, maintaining their continuum of care requires identifying critical barriers outside the health system and within. Making tests available locally is ideal, meanwhile subsidising travel costs, especially for poorer patients, can support the continuum of care over months and years of treatment [48]. The importance of linking access to service delivery was demonstrated in a recent pilot study in Timor-Leste where donor funds were used to build a maternity waiting home next to two CHCs in urban centres, away from Dili. In one evaluation, the initiative was deemed unsuccessful because women from rural areas did not travel there [49]. A systematic review of similar projects in several other countries had mixed results, concluding “the limited uptake of facilities in some settings underlines the need to take account of local customs and practice, and the broader factors facilitating and inhibiting access.” [50]. Persuading the unwell that accessing health services is a positive and safe alternative to traditional medicine at home or no care will require comprehensive improvements in transport systems, staff training and education in professional standards of care, essential for repairing and building patient-community relations [26].

Vivid descriptions of patients’ and their companions’ determined efforts to reach hospital, confronting challenging physical conditions, expending limited funds and borrowing more, and testing established cultural boundaries to travel far from home to visit hospital, each demonstrate a profound commitment by these families to achieve the best health outcome possible. What the research also shows is that this commitment is being undermined by physical, financial, operational and socio-cultural barriers, preventing or dissuading many from seeking care, especially the more vulnerable. Poverty and lack of infrastructure (particularly transport), combined with a weak health system (few service locations, erratic delivery systems, nepotism and uncaring attitudes from staff) and an acceptable alternative to use traditional medicine at home, are all strong determinants of access barriers to hospital care. Despite Timor-Leste having a ‘free’ health system, with out-of-pocket expenditure only 4 % of total health expenditure, wealthier patients continue to utilise hospital services at almost twice the rate of poorer patients [22].

Our focus was to explore community and stakeholder experiences in accessing hospital services and to identify barriers to those services. A conceptual framework based on four well-recognised dimensions of access (availability, accessibility, affordability and acceptability) was used. The barriers identified under each dimension were not mutually exclusive. For example, limited access to patient transport directly impacts out-of-pocket expenses (paying for alternative transport and repatriating the deceased), increased risk to the patient’s health (travelling in ill-equipped transport without medical supervision) and delayed care (assuming good quality patient transport is the most efficient means of transport). This is consistent with the work of others such as James et al. [51] and Patcharanarumol et al. [52] who also report important interactions between access barriers. More research is needed to understand the contextual factors that facilitate and undermine the success of interventions in different settings. Studies consistently show that similar interventions to address access barriers in slightly different environments can produce diverse results; understanding local nuances and how they might impact policy initiatives is essential to achieve optimum outcomes [53, 54].

Our study design, using qualitative research and conceptual framework, focuses on social attitudes and personal experiences of the health system. It reveals a complex web of factors influencing both the demand and supply for hospital services in Timor-Leste. Currently small pilot evaluations of individual demand side or supply side interventions are the norm. However, findings from this study point to the need for government and donor stakeholders to develop a more holistic understanding of the barriers to hospital care before appropriate interventions be put in place. Interventions informed by careful assessment of local constraints must be evaluated across wider geographic areas, with attention to implementation requirements (eligibility, incentives, potential for informal payments and corruption), the impact of incentives on user and supplier behaviour and sustainabilty [52, 53].

## Conclusions

This study has demonstrated that having a ‘free’ public health system funded by government is an enormous step towards universal coverage but it is not sufficient to ensure health services are accessible. We have identified both supply and demand side barriers that need to be remedied to improve access to hospital services in Timor-Leste. On the supply side, the shortages in patient transport, medicines, blood supplies, laboratory testing and health workers’ attitudes to the poor need to be addressed. They discourage greater utilisation of the available services. On the demand side, the indirect costs associated with the use of hospital services, such as transport to and from health facilities, accommodation and food for accompanying relatives, and private transport to return the deceased home for burial, nepotism and denigrating staff behaviour equally deserve attention. There are several policy initiatives underway in Timor-Leste to strengthen the health system including improving the ambulance service and developing a stronger pharmaceutical and medical supply management system. Equally important is tackling demand side barriers to encourage patients to seek care, reach the facility and receive patient-centred quality care, and gradually neutralise negative community perceptions and barriers to hospital care.

## Additional files

Additional file 1: Discussion guide for in-depth interviews with Directors of Community Health Centres (CHCS). (DOCX 27 kb) Additional file 2: Topic guide for focus group discussions with community members. (DOCX 22 kb)

## Footnotes

1 The 1999 WHO Expanded Program on Immunization standard vaccine schedule; Bacillus Calmette-Guerin (tuberculosis), diphtheria-tenanus-pertussis, oral polio and measles.

2 Attributions without an age come from one urban female focus group where participant ages were not collected.
