# Supplementary material for: “I go I die, I stay I die, better to stay and die in my house”: understanding the barriers to accessing health care in Timor-Leste
Source: BMC Health Serv Res. 2016 Sep 30;16:535. doi: 10.1186/s12913-016-1762-2 (PMC5045628; doi:10.1186/s12913-016-1762-2)
Supplement: Additional file 1: — Discussion guide for in-depth interviews with Directors of Community Health Centres (CHCS). (DOCX 27 kb) [file 12913_2016_1762_MOESM1_ESM.docx]

**Additional file 1**

**DISCUSSION GUIDE FOR IN-DEPTH INTERVIEWS WITH DIRECTORS OF COMMUNITY HEALTH CENTRES (CHCs)**

**INTRODUCTION:** Good morning/afternoon Director and thank you for consenting to speak with me. My name is ______________ and I would like to hear about your experiences and suggestions for strengthening the hospital system in Timor Leste. In particular we want to understand how patients are referred from your CHC to hospital and any challenges that this might involve. No names will be attached to any statement so feel free to share your views. The reason why we are doing this is to help the government/ MOH improve access to hospital services especially for the poor. The interview should take less than 1 hour and will be tape recorded because we don’t want to miss any of your comments. Do you have any questions before we begin?

**BACKGROUND**

**1. What do you think about hospital services in Timor-Leste, in general?**

**Possible areas to probe:**

- Easy accessibility (can everybody get access, opening hours, do patients come from outside your catchment area – if so, why?)
- Affordability (no fees, out-of-pocket expenses)
- Services of reasonable quality (main services provided, in-patient care, adequate equipment)

**REFERRAL SYSTEM**

**2. What are the main reasons that patients are referred to a hospital from your CHC?**

**Possible areas to probe:**

- Frequency (are patients referred from this CHC to hospital?)
- Destination (are patients referred to the regional hospital or to Dili national hospital?)
- Main reasons for referral (is referral usually for diagnosis or treatment, or for both?)

**3. Do you have written guidelines about how to refer patients?**

**Possible areas to probe:**

- Understanding (do the staff understand the referral guidelines?)
- Implementation (do the staff know how to implement the guidelines?)
- Necessity (do you think it is necessary to have written guidelines?)

**4. How do patients and their families react when you refer them to hospital?**

**Possible areas to probe:**

- Utilisation (do patients use the referral?)
- If NO – do they give a reason why?

**5. What challenges do you and patients face when referred to hospital?**

**Possible areas to probe:**

- Challenges (obstacles that prevent you from referring patients to hospital)
- Suggestions (how can the referral system be improved for both staff and patients?)

**6. Do you have a system for monitoring the patients once they get out of hospital?**

**Possible areas to probe:**

- Follow-up visits (do patients return to the CHC after they are released from hospital?)

**7. If patients do not use their referral to hospital, what do you think they do instead?**

**Possible areas to probe:**

- Traditional medicine (do patients choose traditional medicine instead?)

**ACCESS TO HOSPITAL SERVICES**

**8 What do you think are the main barriers that patient’s perceive/face when they need to go to hospital?**

**Possible areas to probe:**

- Transport availability (patients always have access to transport to get to (and from) hospital)
- Costs (do patients spend any of their own money when they are referred to hospital?)
- Services availability (are medicines and tests available at hospitals?)
- Professional standards (are patients treated equally and with respect?)

**9.** **Are some patients more likely to be referred than others?**

**Possible areas to probe:**

- Nepotism (do poor people, people from rural areas, or people with no connections at the hospital have difficulties accessing hospital care in Timor-Leste?)

**CONCLUSION**

**10. What do you think can be done to improve access to hospital services, especially for the poor?**

- Suggestions (to limit nepotism, any other suggestions)

**THANKS** Thank you for taking the time to speak with us today and for your insights. It is very much appreciated and it will be an important contribution to our study.
