# Supplementary material for: “I go I die, I stay I die, better to stay and die in my house”: understanding the barriers to accessing health care in Timor-Leste
Source: BMC Health Serv Res. 2016 Sep 30;16:535. doi: 10.1186/s12913-016-1762-2 (PMC5045628; doi:10.1186/s12913-016-1762-2)
Supplement: Additional file 2: — Topic guide for focus group discussions with community members. (DOCX 22 kb) [file 12913_2016_1762_MOESM2_ESM.docx]

**Additional file 2**

**TOPIC GUIDE FOR FOCUS GROUP DISCUSSIONS WITH COMMUNITY MEMBERS**

**INTRODUCTION**: Good morning/afternoon everyone and thank you for agreeing to join today’s focus group discussion and submitting your written consent. My name is ______________ and I will facilitate the discussion about the use of hospital services in this country – both public and private hospitals. We want your views on how people use hospital services and what may prevent people from using these services. It is a general discussion so nobody will be identified as providing any specific information. No names will be attached to any statement, so feel free to share your views. The reason why we are doing this is to help the government/ MOH improve access to hospital services, especially for the poor. The discussion should take less than 1 hour and will be tape recorded because we don’t want to miss any of your comments. Do you have any questions before we begin?

| 1. **What do you think about hospital services in Timor Leste, in general?**   **Possible areas to probe:**   - Easy accessibility (can everybody get access?) - Affordable (no fees) - Quality of services (health worker attitude, waiting time, availability of medicines, etc.)  1. **Have you or any member of your household ever used hospital services?**   **Possible areas to probe:**   - For YES - Purpose of usage: deliveries, chronic disease management, accident and emergencies, etc. - For NO – Barriers to using hospital services: costs, distance, transport, no need, referrals, alternative medicines, etc.   *(EXPERIENCES/PERCEPTIONS OF PEOPLE IMPORTANT!)*   1. **Sometimes when you attend a clinic, the doctor/health worker there will have to refer you to a hospital for further treatment. Do you think people (including members of your household) are able to get a referral to use hospital services when needed?**   **Possible areas to probe:**   - Experiences/perceptions of people getting hospital referrals - Knowledge of people using or not using hospital referrals when given - Overseas referrals  1. **What do you think are some of the difficulties households face in using hospital services?**   **Possible areas to probe:**   - Costs - Distance - Transport, etc. - Attitudes of staff  1. **What do you think can be done to improve access to hospital services, especially for the poor?** |
| --- |
